# Real-time suicide surveillance supporting policy and practice

**DOI:** 10.1017/gmh.2022.42

**Published:** 2022-08-12

**Authors:** R. Benson, C. Brunsdon, J. Rigby, P. Corcoran, M. Ryan, E. Cassidy, P. Dodd, D. Hennebry, E. Arensman

**Affiliations:** 1 School of Public Health, College of Medicine and Health, University College Cork, Cork, Ireland; 2 National Suicide Research Foundation, WHO Collaborating Centre for Surveillance and Research in Suicide Prevention, Cork, Ireland; 3 National Centre for Geocomputation, National University of Ireland Maynooth, Maynooth, Ireland; 4 Cork Kerry Community Health Services, Health Service Executive, Cork, Ireland; 5 Department of Psychiatry and Neurobehavioural Science, University College Cork, Cork, Ireland; 6 National Office for Suicide Prevention, Health Service Executive, Dublin, Ireland; 7 Australian Institute for Suicide Research and Prevention, School of Applied Psychology, Griffith University, Brisbane, Australia

**Keywords:** climate change, crisis response, humanitarian crises, real-time suicide surveillance, public health emergencies

## Abstract

Suicide mortality rates are a strong indicator of population mental-health and can be used to determine the efficacy of prevention measures. Monitoring suicide mortality rates in real-time provides an evidence-base to inform targeted interventions in a timely manner and accelerate suicide prevention responses. This paper outlines the importance of real-time suicide surveillance in the context of policy and practice, with a particular focus on public health and humanitarian crises.

The suicide mortality rate of a country is a macro-indicator of the wellbeing and mental health and offers a baseline for evaluation of suicide prevention programme efficacy (Värnik *et al*., 2012). However, the quality of suicide mortality data varies on a global level due to stigma, limited resources, and misclassification in some countries where suicide is deemed a criminal offence (Mishara and Weisstub, 2016). Furthermore, complex death investigations involving multiple parties result in delays in the release of official national suicide mortality statistics averaging around two years (Ikeda *et al*., 2014). These issues impact on the timeliness, accuracy and reliability of suicide mortality data and hinder comparative analysis between countries (Värnik *et al*., 2010; Platt *et al*., 2019).

The implementation of real-time suicide surveillance could greatly benefit suicide prevention strategies and policies. The World Health Organization (WHO) has repeatedly promoted the value of real-time surveillance frameworks for mental health issues including suicide and has emphasised the need for reliable and timely data to inform decision-making, to determine matters requiring immediate action and to facilitate the evaluation of intervention and prevention programmes (WHO, 2018, 2019*a*). Global policy outlined in the WHO Comprehensive Mental Health Action Plan 2013–2030 includes an objective to strengthen information systems, evidence, and research for mental health (WHO, 2021*a*), building on the WHO Global Report ‘Preventing Suicide: A Global Imperative’, which signals the need for an increase in the quality and timeliness of national data on suicide and suicide attempts (WHO, 2014, p. 57). Moreover, the United Nations (UN) Sustainable Development Goals target 3.4 calls for a reduction in premature mortality from non-communicable diseases by one third by 2030, identifying the suicide mortality rate per 100 000 people as an indicator of progress (UN, 2015), thus relying on timely monitoring of suicide rates internationally to assess the efficacy of prevention strategies.

The release of the WHO ‘Live Life: an implementation guide for suicide prevention in countries’ provides a comprehensive framework to support countries in developing and implementing national suicide prevention strategies (WHO, 2021*b*). A core pillar of the guide is ‘surveillance, monitoring, and evaluation’, with a strong emphasis on publishing data based on rates and trends regularly to inform action towards reducing overall rates of suicidal behaviour. Without this information, it is difficult to measure progress towards key components of a national suicide prevention strategy. The interventions listed in the guide, particularly means-restriction, media liaison regarding responsible reporting, as well as early identification, assessment, management, and follow-up of those affected by suicidal behaviour all depend on the availability of real-time suicide surveillance to facilitate and direct such actions.

## Real-time suicide surveillance facilitating the identification of emerging suicide clusters

While broad aggregate-level data is important for the implementation of national strategies, small area-level data is essential to local suicide prevention efforts, of which the use of spatial epidemiological methods can increase the reliability of such data (Bommersbach *et al*., 2012; Torok *et al*., 2019). Furthermore, community responses to suicide clusters benefit from using such surveillance in pre- and post-intervention evaluation to determine its efficacy in mitigating further suicide contagion (Lai *et al*., 2020). Investigations of this kind, based on real-time suicide data, would serve to facilitate earlier detection of suicide clusters and timely responses in affected communities (Hawton *et al*., 2015, 2020; Health Service Executive, 2021). The data would also facilitate the reallocation of resources to impacted areas, communities, and settings, while geospatial analysis can effectively identify geographical areas affected by recurring suicide clusters, which require a more intensive and targeted response.

Given that community perception of a suicide cluster has been found to contribute to the potential contagious effects (O'Carroll and Mercy, 1990), verification of speculative rumours of suicide clustering in a community is a critical feature of a real-time suicide surveillance system (Knipe *et al*., 2021). Inappropriate and inaccurate reporting of suicide in the media can lead to sensationalism based on unfounded claims. While the data collated by a real-time suicide surveillance system is provisional given the immediacy of data entry, the availability of up-to-date information on suspected suicide cases provides an accurate source that can be used to validate unconfirmed reports of emerging trends. As a result, media professionals may refer to such a system to verify unsubstantiated statements prior to publication or modification of original published reports, thereby avoiding unwarranted community concern and negative escalating effects. This specific feature of real-time suicide surveillance has added value in promoting responsible media reporting of suicide, especially in the current era of predominantly unregulated, instant information sharing across jurisdictions via social media and contemporary communication technologies. Furthermore, media coverage of a high-profile case of suicide has been linked with an increase of between 8% and 18% in the 1–2 months following the media reports and an increase of 30% of cases by the same method of suicide wherein details of the method of suicide were reported (Niederkrotenthaler *et al*., 2020*a*). A real-time suicide surveillance system provides the basis to monitor fluctuations in suicide rates with only a slight lag, facilitating prompt tracking of cases after a particular media report, communication of relevant findings to media personnel, and guidance on subsequent actions (Baran *et al*., 2021).

## Impact of the COVID-19 pandemic on suicide

Media sensationalism of suicide was intensified during the onset of the coronavirus (COVID-19) pandemic in many countries, resulting in the publication of recommendations and guidance for responsible media reporting of suicide in relation to the pandemic (Baran *et al*., 2021; Hawton *et al*., 2021). Availability of recent suicide data to monitor international trends and identify at-risk sub-populations was severely limited in the early stages of the pandemic (Niederkrotenthaler *et al*., 2020*b*), emphasising the urgency for real-time suicide surveillance to provide an up-to-date evidence-base to inform policy responses (Ramchand *et al*., 2021). Existing real-time surveillance systems had the benefit of conducting prompt trend analyses of routinely collated, accurate data (Arensman, 2021; Clapperton *et al*., 2021; Tanaka and Okamoto, 2021), while researchers in some countries searched rigorously on an ad hoc basis to obtain access to data of this type (Pirkis *et al*., 2021), raising concern around the reliability of such data.

Humanitarian crises such as pandemics, climate change and armed conflict are known to impact suicide rates (Bell *et al*., 2012; Burke *et al*., 2018; Sakamoto *et al*., 2021). The confluence of global crises occurring during this unprecedented era and beyond underlines the critical need for practical, systematic, surveillance practices that remain functional during periods of instability to inform crisis response, service planning and preparedness. The ongoing pandemic has demonstrated the rate at which mental health services can be undermined in emergency situations, impacting continuity of care. Crises of such magnitude go above the decision-making and operational capabilities of regular crisis management and require a higher level of preparedness. Rapid action in an emergency is vital; however, it depends on effective contingency planning by mental health services which can be efficiently activated in anticipation of challenges and all eventualities (Conseglieri *et al*., 2021). Such plan development would benefit from the availability of real-time suicide data to ascertain key areas of focus, while also providing the basis for predictive modelling of suicide rates to inform wider mental health and suicide prevention policies.

## Impact of conflict and war on suicide among refugees

In the third year of this ongoing global pandemic, the sudden invasion by Russia on Ukraine has plunged the world into a renewed catastrophe. Significant armed conflict has engulfed the nation, forcing millions of civilians to immediately evacuate their native land to escape war. The mass exodus of abruptly displaced individuals resulting from this evolving crisis adds to an already record global level of internally displaced individuals, refugees, and asylum seekers that reached 82.4 million at the end of 2020 and will undoubtedly continue to dramatically increase over the coming months and years (UN High Commissioner for Refugees, 2021). The most recently recorded suicide mortality statistics in both Russia and Ukraine indicate that both countries rank among the highest suicide rates globally, even prior to the recent outbreak of armed conflict. Furthermore, nearby host countries of those escaping the war in Ukraine, such as Lithuania, Slovenia, and Hungary report the highest recorded suicide rates in Europe (WHO, 2021*c*). The concerning rates in those countries emphasise the need to monitor suicide rates in as timely a manner as possible to identify possible further increases stemming from the conflict and migration crises.

Individuals escaping conflict are at an elevated risk of experiencing a range of adverse mental health outcomes, resulting from exposure to extreme trauma (Steel *et al*., 2009; Silove *et al*., 2017; Blackmore *et al*., 2020). Despite the extensive evidence documenting increased vulnerability among those who experience armed conflict, humanitarian response efforts often overlook the mental health impacts and the necessity of psychosocial support for those affected (International Federation of Red Cross and Red Crescent Societies Reference Centre for Psychosocial Support, 2021). Displaced populations are understood to be at an elevated risk of suicide, especially those from countries with increased suicide risk, and particularly during the acute phase (Spallek *et al*., 2015; Forte *et al*., 2018; Haroz *et al*., 2020). Yet, data on suicide in refugees is both lacking and inconclusive, with most statistics based on high-income countries and limited data available for low- and middle-income countries who host approximately 85% of the globally displaced population (Ager *et al*., 2021). Research has identified that the psychological distress experienced by displaced individuals is not the sole cause of exposure to war-related violence. It is also strongly linked with displacement-related stressors including social isolation, unemployment, poverty, and perceived discrimination. Furthermore, such distress is compounded by issues including uncertainty surrounding legal status, the threat of deportation or lengthy periods stuck in detention centres for asylum seekers (Miller and Rasmussen, 2017). A major gap exists in the literature to address the efficacy of interventions addressing such stressors, reaffirming the need to obtain current data to respond effectively to the mental health challenges affecting vulnerable subpopulations both in the short-term and longer-term. Moreover, research investigating suicide prevention and responses to refugees is significantly sparse (Haroz *et al*., 2020). Together, these issues provide a strong argument for the real-time surveillance of suicide mortality as a tool for advocacy which can support host countries in developing policy and strengthening mental health services to provide the essential components of timely diagnosis and treatment for mental illnesses, aligned with the objectives of the WHO Draft Global Action Plan ‘Promoting the Health of Refugees and Migrants’ 2019–2023 (WHO, 2019*b*).

## Impact of climate change on suicide

The climate crisis poses an increasing environmental threat to humanity, triggering climate and eco-anxiety in the global population, particularly among children and young people (Taylor, 2020; Hickman *et al*., 2021). Research based on global warming has revealed a link between rising temperatures and suicide mortality, a trend projected to continue in line with increasing temperature levels (Burke *et al*., 2018), warranting ongoing surveillance using real-time data to detect sudden changes. Natural disasters are also known to impact suicide rates, with fluctuations observed in affected populations both in the direct aftermath and recovery phases (Kõlves *et al*., 2013; Horney *et al*., 2020; Orui, 2020). Routinely collected suicide mortality can be used to inform immediate and long-term post-disaster suicide prevention responses to support those directly affected.

Seasonal variation in suicide rates have been detected, hypothesised to be the result of factors such as weather-related changes or shifts in social interaction (Bando and Volpe, 2014; Fester, 2021). Real-time suicide surveillance lends itself to trend analysis, through which seasonal variation, emerging trends, and high-risk periods can be detected in real-time, providing the opportunity for early intervention. While providing an evidence-base for suicide prevention policy, the collation of real-time suicide data also serves to inform and support climate change and environmental-based policies by providing an insight into the influences of climate-related stressors on mental-health and suicidality by way of lobbying governments to act to reverse the harmful effects on the climate.

## Impact of concurrent public health emergencies on suicide

The concurrence of armed conflict, a pandemic and climate-related stressors, exacerbates the stressors experienced by individuals, resulting in increased anxiety and linked disorders such as anxiety disorders, in particular PTSD, which may lead to increased suicide risk. The link between disease outbreak and climate change indicates that future pandemics are unavoidable (Taylor, 2019). While evidence indicates that climate change was not the root cause of the current COVID-19 pandemic, global warming is a known risk factor for the spread of infectious diseases such as tropical disease outbreaks in wetter, warming climates that offer optimal environments for disease vectors (Taylor, 2020). Moreover, pandemics can escalate conflict risks through factors such as increased grievances, altered opportunity factors for organised armed groups and deteriorating levels of democracy (Ide, 2021).

The recently observed rise in armed conflict in some parts of the world is a concerning trend given the threat of combat to human safety, as well as the capacity of countries to deal with a public health crisis during warfare. These concerns are further heightened by the anticipated adverse economic impact of these co-occurring crises on suicide rates, particularly given the risk of financial recession stemming from armed conflict and public health emergencies independently, together with the existing suicide risk directly associated with economic crisis (Oyesanya *et al*., 2015). The long-term effects of the present turbulent period should be considered and planned for in advance (Sinyor *et al*., 2021), making use of real-time data to inform targeted strategies that would ease the lasting impact on mental health and prevent suicide. The rapidity and simultaneity of these humanitarian crises should provide the impetus for governments to ensure their readiness to respond efficiently to future crises. It is evidently clear that now, more than ever, there is a valid requirement for real-time suicide surveillance to monitor the evolving situation and beyond, with the fundamental purpose to reduce suicide.

The implementation of recommended components and practices applied by established real-time suicide surveillance systems elsewhere in the world, while adapting to regional/local circumstances and resource availability, would support the development of real-time suicide surveillance. Networking and knowledge exchange would further facilitate the development of real-time suicide surveillance systems through a learning process from peers with both experience and expertise in the innovative area of research (Baran *et al*., 2021).
